# Expected Impact of the Pass/Fail Scoring System for USMLE Step 1 on the Plastic Surgery Residency Selection Process: A National Survey of Plastics Program Directors

**DOI:** 10.7759/cureus.29411

**Published:** 2022-09-21

**Authors:** Michael S Powell, Laila L Rhodes, Santiago Gonzalez, Sagar T Mehta

**Affiliations:** 1 College of Medicine, University of Arkansas for Medical Sciences, Little Rock, USA; 2 Critical Care, The Ohio State University College of Public Health, Columbus, USA; 3 Division of Plastic and Reconstructive Surgery, University of California San Francisco, San Francisco, USA; 4 Division of Plastic and Reconstructive Surgery, University of Arkansas for Medical Sciences, Little Rock, USA

**Keywords:** national survey, residency program director, plastic surgery residency, step 2, usmle step 1 pass/fail

## Abstract

Background: Traditionally, the United States Medical Licensing Examination (USMLE) Step 1 3-digit score has been used as a metric to stratify plastic surgery residency candidates. The transition to a pass/fail exam may impact the manner in which integrated plastic surgery residency program directors (PS-RPD) evaluate candidates. It may also limit opportunities for applicants to differentiate themselves from their counterparts.

Methods: A 14-question survey was distributed via email to 76 PS-RPDs collected from the American Medical Association (AMA) residency program site, FRIEDA. It was sent three times from March 3 - March 14, 2020. McNemar tests were performed on the current metrics of evaluation in comparison to metrics expected to be used in the absence of a 3-digit Step 1 score, assuming a P < 0.05 level for statistical significance.

Results: Of the 76 integrated plastics programs surveyed, 24 PS-RPDs responded (31.6% response rate); 91.3% of PS-RPDs strongly disagree or disagree that Step 1 should be pass/fail; 78.3% of PS-RPDs strongly disagree or disagree that diversity will increase. The top five evaluation metrics PS-RPDs expect to utilize following the transition to pass/fail are: letters of recommendation (87.0%; CI 72% - 100%; p=0.500), Step 2 score (78.3%; CI 60% - 96%; p=0.001), research (56.5%; CI 35% - 78%; p=0.125), elective rotation (56.5%; CI 35% - 78%; p=1.000), and personal knowledge of the applicant (52.2%; CI 30% - 74%; p=0.500).

Conclusions: In the absence of a Step 1 score, PS-RPDs may require more holistic metric(s) to evaluate the best fit for their program. This study found that PS-RPDs expect their candidate evaluation process to remain highly similar with the only statistically significant change being an increased emphasis on the candidate's Step 2 score.

## Introduction

The United States Medical Licensing Examination (USMLE) Step 1 score is an essential tool used by residency programs to select applicants to interview [1,2]. Officially, the USMLE Step 1 examination has been a metric to gauge if medical students have obtained an appropriate breadth of medical knowledge to become a licensed physician. However, unofficially, the three-digit score has been used as a soft metric for medical students to determine the range of medical specialties that a student can pursue [3-5]. The performance on the USMLE Step 1 is of particular importance to match into competitive fields such as plastic surgery [6,7]. Factors such as Alpha Omega Alpha status, letters of recommendation, and graduating from a top-40 ranked medical school has also been shown to increase the chances to match into an integrated plastic surgery residency [8]. Despite the variety of factors that may play a role in an applicant’s likelihood of matching, it is clear that performance on the USMLE Step 1 is of utmost importance [8-11]. As a result, it is reasonable to expect that any modification to the exam’s structure or grading system may have a significant impact on the plastic surgery residency selection process. 

On February 12, 2020, the National Board of Medical Examiners (NBME) and the Federation of State Medical Boards (FSMB) announced that the USMLE Step 1 will change its grading system from a three-digit numeric score to a pass/fail grade [12]. This transition took effect in January 2022, which may impact the plastic surgery residency selection process for applicants as early as the 2023 application cycle. Previous studies found that there are mixed thoughts about the change in the USMLE Step 1 scoring system [13-15].

In this study, we surveyed integrated plastic surgery program directors from programs across the United States as a means to better understand how candidate evaluation is expected to change secondary to USMLE Step 1 becoming pass/fail. This is meant to be a resource for future applicants as they strive to maximize their chances of successfully matching into a plastic surgery residency position. 

## Materials and methods

A total of 82 PS-RPDs’ email addresses were obtained from the American Medical Association (AMA) residency program site FRIEDA. Of the 82 residency programs, six of the email addresses were returned as undeliverable. The correspondence began on March 2, 2020 with an invitation email to all 82 programs. Three subsequent survey invitations were sent to the programs between March 3 and March 9, 2020. PS-RPDs who elected to participate utilized a link provided to the REDcap survey which remained unlinked to their email to preserve complete program directors’ anonymity. After completion of the survey, program directors were able to view a graphic summary of the survey data collected until it was closed on March 14, 2020, at 12 pm CST. Refer to the Appendices for the survey questions.

In order to identify which metrics will become more important during future candidate evaluation, program directors were provided with a list of 18 metrics based on the program directors’ survey from the National Residency Match Program [1]. The results of the study were collected using REDCap electronic data capture tools hosted at the University of Arkansas for Medical Sciences and the data were de-identified prior to statistical analysis [16,17]. The study was approved using exempt procedures by the Institutional Review Board at the University of Arkansas for Medical Sciences prior to the dissemination of the survey (IRB# 260779). As a part of the survey, participants were informed that their responses could be used for research purposes.

Statistical software SPSS version 26 (IBM Corp., Armonk, NY) was used to analyze the qualitative and quantitative data. Frequency distribution was considered for all survey questions to find evidence of outliers and potentially statistically significant information. Descriptive statistics were used to summarize participant responses as relevant to the objective and further elucidate the characteristics of the programs that participated in the study. Cross tabulation of current evaluation metrics with expected evaluation metrics was used to describe how PS-RPDs foresee the new pass/fail system impacting candidate evaluation. McNemar tests were performed on the current metrics of evaluation in comparison to metrics expected to be used in the absence of a 3-digit Step 1 score. 

## Results

Demographics

Of the respondents (n=24), 79.2% represent university-based plastic surgery residency programs. The respondents represent a wide range of experience as PS-RPDs with 41.7% having 0-5 years of experience, 25% having 5-10 years of experience, and 33.3% having over 10 years of experience (Table 1). For a full summary of the collected respondents’ demographic information, refer to Table 1.

**Table 1:** Demographics of Plastic Surgery Residency Program Director Respondents (n=24)

	Survey Respondents	% of survey respondents	Total Number of programs
Respondents Program Type			
University based	19	79.2%	63
Community based	0	0.0%	3
Community based/ University affiliated	5	20.8%	16
Total Programs	24	100.0%	82*
Number of years as Program Director			
0-5 years experience	10	41.7%	
5-10 years experience	6	25.0%	
>10 years experience	8	33.3%	
Uses of Step 1 as screening tool			
Does not use Step 1 as a screening tool	7	29.2%	
Between 194-204	0	0.0%	
Between 205-214	0	0.0%	
Between 215-224	2	8.3%	
Between 225-234	3	12.5%	
Between 235-244	7	29.2%	
Between 245-254	4	16.7%	
* Total number of integrated plastic surgery residency programs was collected from https://freida.ama-assn.org/. 6 of the 82 programs did not receive the survey because the survey invitation was returned as "undeliverable".			

Step 1 as a screening tool

In the evaluation of plastic surgery residency candidates, Step 1 is largely utilized as a cutoff metric to screen the large number of applicants applying for a coveted plastic surgery residency positions [6,7]; 66.7% of respondents to this study report their program currently utilizes the Step 1 score as a screening tool. The most common screening score range was reported as, “between 235-244” (29.2%) (Table 1).

Attitudes Towards Step 1 Becoming Pass/Fail

When asked the extent to which they agree/disagree with Step 1 becoming pass/fail, 78.3% strongly disagree and an additional 13.0% disagree with the change in scoring systems. No PS-RPDs among the cohort agree with Step 1 becoming pass/fail. The change in scoring system has the potential for unintended consequences which the PR-RPS’s highlighted by offering the following responses:

“Unfortunately we will ultimately not be able to tell which medical students are the hardest working, most motivated achievers in medical school.” (R1026)

“Placing more less qualified residents in subspecialties that requires higher level functions. Therefore decreasing the overall quality of the end product of these specialties and patients will suffer for it.” (R0477)

“More resources [will be] required by program staff who are already overworked” (R0487)

Diversity Among Plastic Surgery Residents

**Figure 1:**
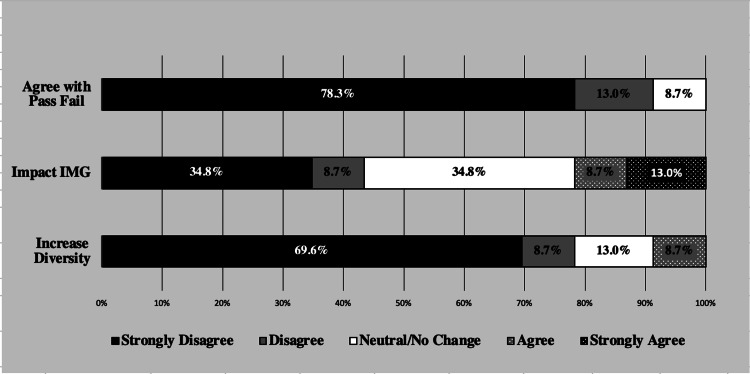
Plastic Surgery Residency Program Director Likert Responses (n=23)

USMLE states that “maintaining 3-digit score reporting on the Step 1 exam may limit diversity” [18]. However, among the PS-RPD represented in the study, the majority strongly disagree (69.6%) or disagree (8.7%) with the notion that diversity will increase following Step 1 becoming pass/fail. Only 8.7% of PS-RPD agree that diversity will increase in their program with a transition to a pass/fail exam (Figure 1). While diversity is not explicitly defined by the USMLE, the qualitative data mentions several types of applicants that will be affected:

“The USMLE step 1 was an item that allowed an applicant from a lesser known school, a DO, or a foreign medical grad to be compared against others….Those fortunate to go to the top schools will have a great advantage now that one of the only ways an applicant from a lesser known background could stand out.” (R0445)

Evaluation of Plastic Surgery Residency Candidates

Plastic surgery residency program directors were asked to select their current top five of 1the 8 metrics used to evaluate candidates. Under the current 3-digit USMLE Step 1 scoring system, the most commonly selected metric was Letters of Recommendation (95.7%) followed by Step 1 Score (80.0%), Elective Rotations (56.5%), Grades (43.5%), and Personal Knowledge of Applicant (43.5%) (Table 2). As an instrument to gauge potential weaknesses in the current candidate evaluation system, PS-RPD’s were asked about the primary complaint they receive regarding new residents. Respondents cited clinical knowledge (39.1%) as a more common complaint than basic science knowledge (17.4%).

**Table 2:** Expected Top Five Metrics - Plastic and Reconstructive Surgery (n=23)

Metric	Current Top 5 Metrics	Expected Top 5 Metric	P-value
	% respondents select as Top 5 metric (CI 95%)		
Step 1	87% (72% - 100%)	N/A	N/A
Letters of Recommendation	95.7% (87% - 100%)	87% (72% - 100%)	0.500
Step 2	30.4% (10% - 51%)	78.3% (60% - 96%)	0.001
Research	39.1% (18% - 61%)	56.5% (35% - 78%)	0.125
Elective Rotation	56.5% (35% - 78%)	56.5% (35% - 78%)	1.000
Personal knowledge	43.5% (22% - 65%)	52.2% (30% - 74%)	0.500
Class Rank	26.1% (7% - 46%)	34.8% (14% - 56%)	0.500
Grades	43.5% (22% - 65%)	30.4% (10% - 51%)	0.250
Deans Letter	8.7% (0% - 21%)	21.7% (4% - 40%)	0.250
Medical School	8.7% (0% - 21%)	17.4% (1% - 34%)	0.500
AOA	17.4% (1% - 34%)	17.4% (1% - 34%)	1.000
Honors/Awards	8.7% (0% - 21%)	13% (0% - 28%)	1.000
Leadership	21.7% (4% - 40%)	13% (0% - 28%)	0.500
Personal Statement	4.3% (0% - 13%)	8.7% (0% -21%)	1.000
Extracurricular	4.3% (0% - 13%)	4.3% (0% - 13%)	1.000
Volunteer	0.0% (N/A)	4.3% (0% -13%)	N/A
Local Connections	8.7% (0% - 21%)	4.3% (0% -13%)	1.000
Post Grad Degree	0.0% (N/A)	0.0% (N/A)	(N/A)

Program directors were then asked to select their expected top five out of 17 metrics to be used to evaluate candidates with the new pass/fail system. The expected top five weighted metrics are; letters of recommendation (87.0%; CI 72% - 100%; p=0.500), step 2 score (78.3%; CI 60% - 96%; p=0.001), research (56.5%; CI 35% - 78%; p=0.125), elective rotation (56.5%; CI 35% - 78%; p=1.000), and personal knowledge of the applicant (52.2%; CI 30% - 74%; p=0.500) (Table 2). Additionally, when assessing the open-ended responses, PS-RPDs emphasized the increased value of Step 2 scores and a possible need for candidates to complete the Step 2 examination earlier. Statements discussing the expected adjustment in the importance of each metrics under the new system include:

“We will use Step 2 scores instead of Step 1 scores as we need a mechanism to be able to select candidates from hundreds of applicants.” (R0549)

“I assume that many programs will use Step 2 scores to screen applicants.” (R0447)

Transition to New Candidate Evaluation Metrics

The USMLE announced in February, 2020 their intention to introduce the new Step 1 pass/fail scoring system beginning in January, 2022 [2]. The delayed implementation of the new scoring system prompts the question of when PS-RPDs expect to adjust their use of a Step 1 score for candidate evaluation. 78.3% of PS-RPD respondents report they are unlikely to make changes in candidate evaluation until Step 1 is no longer reported as a 3-digit score.

## Discussion

PS-RPDs are faced with the challenge of evaluating a high volume of residency candidate applications quickly and thoroughly. Of PS-RPDs in this study, 66.7% use Step 1 as a cut-off metric to screen candidates for interviews (Table 1). When asked about the change in the scoring system, 91.3% of PS-RPDs disagree that Step 1 should be a pass/fail exam (Figure 1).

In the absence of a 3-digit Step 1 score, the candidate evaluation metrics which PS-RPDs expected to become most utilized are; Letters of recommendation, Step 2 score, and Research (Table 2). The most statistically significant change in metric between the current evaluation system and the expected evaluation system is the utilization of a Step 2 score (30.4% - 78.3%; p=0.001) (Table 2). The expected shift suggests PS-RPDs are attempting to identify another quantitative metric to replace the 3-digit Step 1 score.

Both quantitative and qualitative data from this study suggest that a candidate’s Step 2 score will become a pivotal component of the program’s new candidate evaluation system. With an increased emphasis on Step 2 as a metric in candidate selection, program directors may be selecting residents with better clinical knowledge. This may inadvertently improve the current deficit in clinical knowledge reported by 39.1% of PS-RPDs. Further investigation is warranted to determine if the reported lack of residents’ clinical knowledge improves with an increased emphasis on Step 2 scores when evaluating resident candidates.

The USMLE posed the concern that an over emphasis on Step 1 scores may impact the diversity of individuals interviewed and ultimately selected for a residency program. To the credit of integrated plastic surgery programs in the US, there have been significant improvements in gender equality among plastics residents [19]. But racial and ethnic diversity of plastics residents decreased from 2010-2016, “Despite an increase in integrated applicant proportions, the Black resident proportion is decreasing” [19]. As the root cause(s) for the lack of racial and ethnic diversity among plastic residents continues to be explored; this study found that 78.3% of PS-RPDs disagree that Step 1 as a pass/fail exam will result in increased diversity among integrated plastic surgery residents. Perhaps Step 1 becoming pass/fail will provide the catalyst for PS-RPDs to utilize their innovative natures as plastic surgeons to revisit the candidate evaluation processes to find new ways to address the current diversity among current board-certified plastic surgeons.

The pedigree of the medical school a candidate attended is a traditional metric in the residency candidate evaluation process. The qualitative data from this study suggest the removal of a 3-digit Step 1 score will further benefit top medical school graduates. This will put candidates from lesser-known programs at a distinct disadvantage in the absence of a Step 1 score to quantitatively demonstrate their academic capacity. The USMLE suggests that Step 1 becoming pass/fail will remove a barrier that is currently impeding diversity among residents [18]. But the data from this study suggest candidates who do not attend a top medical school will be at a disadvantage as a result of the change in Step 1 scoring - specifically, candidates from IMG, DO programs, and lesser-known MD programs. Additionally, it could be reasonably posited that an increased emphasis on a residency candidate's pedigree will result in integrated plastics residency programs mirroring the diversity represented in top medical schools. 

With USMLE announcing two years before the implementation of a pass/fail Step 1 exam it provided an opportunity for program directors to transition their evaluation by gradually decreasing their weighting of 3-digit Step 1 score as a metric. However, PS-RPDs widely agree (78.3%) that they are unlikely to adjust their current evaluation process until candidates no longer apply with a 3-digit score. This transition phase particularly impacts candidates who may receive a 3-digit score, but will apply to residency with other applicants that received a pass/fail score. As the unprecedented shift in Step 1 scoring continues to develop, it will likely leave medical students unsure of how best to differentiate themselves from their peers.

Some limitations of this study are the inherent challenge that some program directors may be more or less inclined to respond, resulting in the potential for nonresponse bias. Additionally, this survey was conducted shortly after the USMLE announced the change in the scoring system which has provided the opportunity for program directors to develop new systems to adjust to the challenges of evaluating applicants.

## Conclusions

The need to evaluate residency candidates holistically is not disputed. However, in the absence of a 3-digit Step 1 score, PS-RPDs will have less measured criteria to evaluate candidates to ensure the best fit between candidates and programs. This study demonstrates that 91.3% of PS-RPD disagree that Step 1 should be a pass/fail exam and they subsequently expect to increase their utilization of an applicant's Step 2 score (30.4% - 78.3%; p=0.001). Although the USMLE Step 1 pass/fail scoring system may ensure the appropriate basic science knowledge, the qualitative feedback in this study highlights the scoring change will make it increasingly difficult for highly qualified candidates to differentiate themselves from their peers when applying for an integrated plastic surgery residency position. 

## Figures and Tables

**Figure 2:**
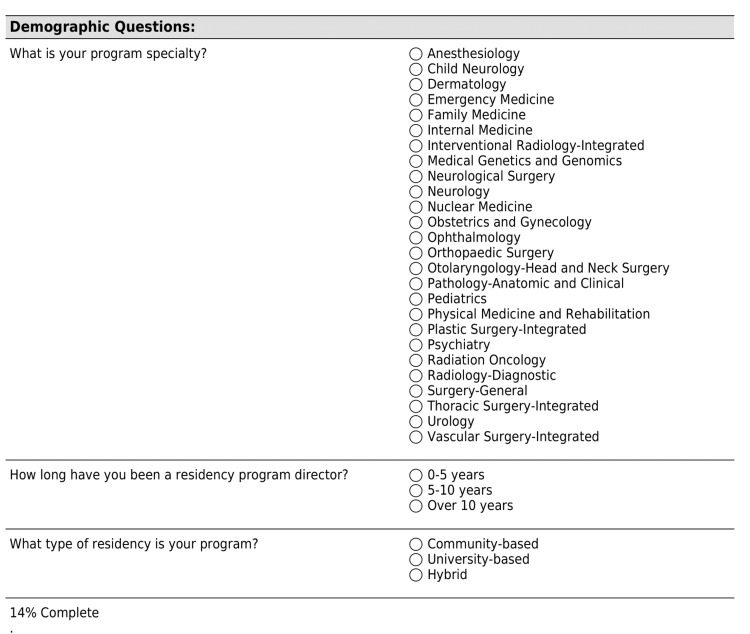
US MD Residency Directors Survey - Page 1

**Figure 3:**
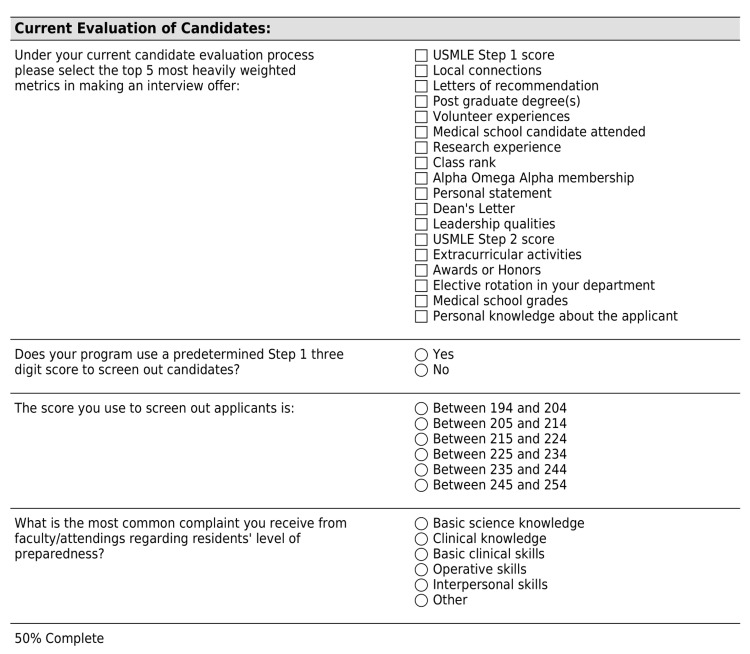
US MD Residency Directors Survey - Page 2

**Figure 4:**
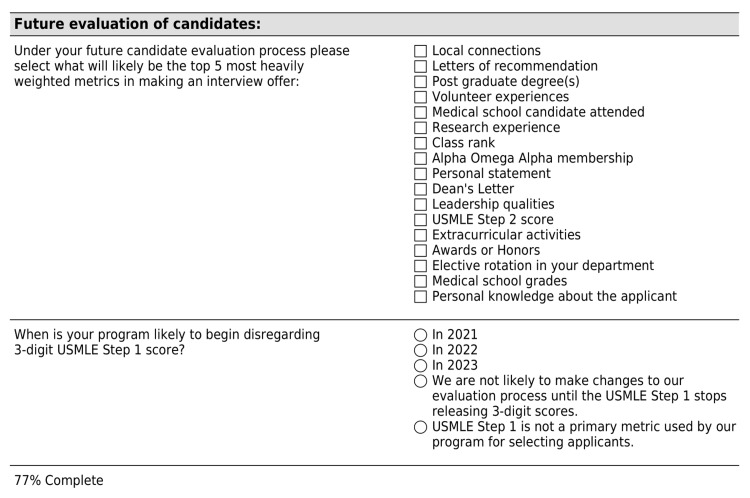
US MD Residency Directors Survey - Page 3

**Figure 5:**
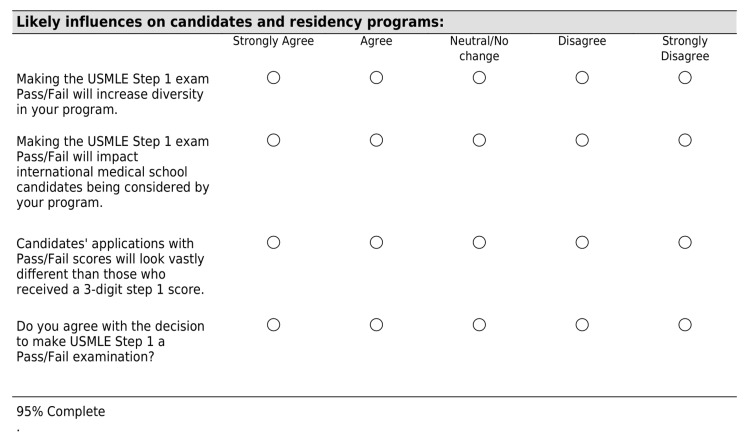
US MD Residency Directors Survey - Page 4

**Figure 6:**
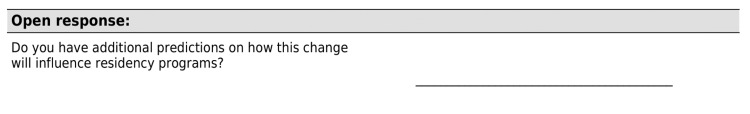
US MD Residency Directors Survey - Page 5
